# The Music-Related Quality of Life (MuRQoL) Questionnaire: Adaptation and Validation into the German Fragebogen zu Musik und Lebensqualität

**DOI:** 10.3390/audiolres16040120

**Published:** 2026-08-17

**Authors:** Nadia Giarbini, Eleonora Catalano, Christian Streitberger, Maria Elena Flacco, Francesco Stomeo

**Affiliations:** 1 Audiology and Phoniatrics Center, HearLife Clinic Bolzano, 39100 Bolzano, Italy; 2 ENT, Audiol and Phoniatrics, Department of Neurosciences and Rehabilitation, University of Ferrara, 44121 Ferrara, Italy; 3 Department of Enviromental and Prevention Sciences, University of Ferrara, 44121 Ferrara, Italy

**Keywords:** music, cochlear implant, music therapy, hearing loss, questionnaire, behavioral measures, instrumentation, psycho-social/emotional

## Abstract

**Purpose:** Growing interest in music perception among cochlear implant (CI) users has led to increasing research in this area. To support cross-cultural studies, this work aimed to adapt and validate the Music-Related Quality of Life (MuRQoL) questionnaire into German (MuRQoL-De). **Methods:** The MuRQoL was translated and culturally adapted into German following established guidelines for hearing-related instruments. The final version (“Fragebogen zu Musik und Lebensqualität”) was completed by 32 adult CI users and 43 normal-hearing (NH) individuals. Internal consistency was assessed using Cronbach’s alpha. Construct validity was evaluated through group comparisons and exploratory regression analyses examining demographic and clinical factors. **Results:** The MuRQoL-De demonstrated high internal consistency, with Cronbach’s alpha values exceeding 0.80 for both Frequency and Importance scales and their subdomains. NH participants reported significantly higher scores than CI users on the Frequency scale, especially in the Music Perception subscale (*p* < 0.01). No significant group differences were observed for the Importance scale. Regression analyses did not identify statistically significant predictors of MuRQoL scores, though trends suggested potential associations with age, education, and musical experience. **Conclusions:** The MuRQoL-De is a reliable and valid instrument for evaluating music-related quality of life in German-speaking adults. It effectively differentiates between CI users and NH individuals in terms of musical participation, while also emphasizing the shared importance of music. Its application may support individualized rehabilitation strategies that include musical engagement, addressing an important but often neglected aspect of auditory well-being.

## 1. Introduction

Cochlear implants (CIs) have reached a high technological standard, making speech understanding with the device an achievable goal for many users [1]. However, most CI recipients still exhibit poor outcomes in music perception [2], which often leads to dissatisfaction with the musical experience and may negatively impact their quality of life (QoL).

Not all patients benefit equally from cochlear implantation, as outcomes are influenced by numerous variables [3]. These include device-specific factors, such as electrode insertion depth, total number of active electrodes and possible anomalies, as well as processing strategy specifications, stimulation mode, current path, loudness levels, and pulse duration. Patient-specific factors such as auditory pathology, neural survival, tissue impedance, and the spatial relationship between electrodes and target neurons also play a significant role [4,5,6].

In addition to these technical and biological determinants, music appraisal, enjoyment, and participation may be shaped by individual expectations, musical background, and personal preferences.

A growing body of evidence supports the idea that music enjoyment positively contributes to QoL, particularly in post-lingually deafened adults [7,8]. Thus, cochlear implantation impacts not only auditory perception and speech-related skills but also broader aspects of social life, personal identity, and emotional well-being [9].

To address these multidimensional needs, the Music-Related Quality of Life (MuRQoL) questionnaire was developed as a tool to guide and evaluate music rehabilitation in adult CI users. It has demonstrated strong psychometric properties, offering a valid and reliable means of assessing music experience and its impact on QoL [10,11].

### 1.1. Music and Quality of Life in Adult CI Users 

Music is a pervasive socio-cultural phenomenon with emotional and cultural significance, fostering social cohesion across the lifespan. Enhancing music appreciation and enjoyment may therefore positively influence quality of life (QoL), social interaction, and perceived benefit from cochlear implantation.

As a routine part of daily life, listening to music can bring joy and improve well-being [12]. Music often gains renewed importance for CI users with postlingual hearing loss once speech perception has been achieved. After implantation, individuals who previously experienced music with normal hearing often express a strong desire to re-engage with and enjoy music [13,14].

However, CI users generally find music less pleasant than normal-hearing (NH) individuals [13]. This was found to be largely due to the limitations of cochlear implant technology in conveying pitch resolution and temporal fine structure, leading to impaired music perception [15]. As a result, adult CI users with postlingual hearing loss often report dissatisfaction with the quality of sound and tend to listen to music less frequently after implantation [13].

From the CI users’ perspective, music remains an important component of emotional well-being and social life [16]. It is therefore disappointing that degraded music perception through CIs restricts access to, and enjoyment of, this meaningful aspect of life [16,17]. Music training may hold clinical relevance by enhancing aspects of auditory perception, such as spectral resolution, that are also important for speech processing. Systematic evaluations of music training programs are necessary to assess their potential benefits for CI users and to better understand the relationship between music engagement and quality of life [3].

Two studies by Lassaletta et al. found a positive relationship between perceived music quality and quality of life in CI users. Specifically, individuals who listened to music more frequently after implantation reported higher QoL scores [7,8,9,10,11,12,13,14]. These findings highlight the importance of music for CI users with postlingual hearing loss and its potential to improve their overall well-being [16]. Despite this, very few studies have directly examined the relationship between music and QoL in adult CI users. Existing questionnaires are not specifically designed to capture music-related experiences or their impact on quality of life. In most prior studies, this relationship has been assessed indirectly, by correlating scores from separate music perception and QoL instruments [18,19].

### 1.2. Current Assessment Tools for Music Experience After Cochlear Implantation

A variety of validated tools currently exist for evaluating music experience in CI users, each offering different perspectives on subjective and/or objective outcomes following implantation. However, to date, no single instrument has gained widespread acceptance for routine clinical or research use. As a result, much of the literature in this field relies on heterogeneous outcome measures, including study-specific or single-use tools, which impedes direct comparison of results across studies and limits the generalizability of findings.

In a recent systematic review, Hwa et al. emphasized this issue, noting that despite a considerable body of research, there remains no standardized tool to comprehensively assess music experience post-implantation [20]. Similarly, Riley et al. highlighted the lack of consensus regarding assessment tools, pointing out their variability in terms of scope, length, clinical feasibility, and frequency of use in the literature. This heterogeneity not only hinders large-scale meta-analyses but also challenges efforts to evaluate the effectiveness of emerging implant technologies and music-based rehabilitation strategies in a consistent manner [21].

Previously reported or validated tools include those listed in the following Table 1:

Music outcomes in CI users have been evaluated through various objective tests, such as the MuSIC test, the Montreal Battery for the Evaluation of Amusia, and the Clinical Assessment of Music Perception. While these tools provide valuable insights into auditory performance, their use remains limited in both clinical and research settings. This is primarily due to the need for specific equipment, trained personnel, and longer administration times compared to conventional audiological assessments. More importantly, the results of such objective tests do not necessarily align with patients’ subjective experiences of music—such as enjoyment, emotional engagement, or social participation [20].

Several music-related questionnaires have been developed for adult CI users, including the Iowa Musical Background Questionnaire (IMBQ), the Munich Music Questionnaire (MUMU), and the University of Canterbury Music Listening Questionnaire (UCMLQ) [16,22,23]. However, these instruments were not designed as standardized tools for assessing rehabilitation outcomes. They often fail to capture important psychosocial dimensions of music perception and engagement, such as emotional responses to music or its role in social interaction, factors that can have a profound impact on QoL. Furthermore, current questionnaires tend to overlook individual differences in musical background, preferences, and expectations, which can substantially influence music appreciation and participation among CI users [10]. As a result, they are often inadequate to evaluate the real-world relevance of music perception and enjoyment in this population.

The absence of a widely accepted, psychometrically robust instrument for assessing music-related outcomes in CI users highlights a significant gap in the field. Addressing this gap is critical for improving our understanding of how music contributes to the auditory and emotional well-being of CI users and for developing targeted interventions that enhance music-related QoL.

### 1.3. The Music-Related Quality of Life Questionnaire (MuRQoL)

The MuRQoL is a relatively recent developed test, reported in 2017 with the explicit aim to assess the impact of music rehabilitation with design for repeated assessment over time [20].

It encompasses two mirror sections, each one containing 18 individual items, one assessing music experiences and the other their importance. Both sections of the questionnaire are divided into two subscales. In the first part (frequency scale), there are 11 items in the “Music Perception” subscale that evaluate the music perception abilities, and 7 items in the “Music Engagement” subscale that evaluate the attitudes towards music and musical activities. In the second part (importance scale), there are 11 items in the “Music Perception” subscale, that evaluate the importance of perception abilities, and 7 items in the “Music Engagement” subscale that evaluate the importance of attitudes towards music and musical activities, which were all questioned in the first part of the questionnaire.

Among the existing music questionnaires designed for adult CI users, the MuRQoL presents several advantages. All items in both sections are measured using a uniform five-point Likert scale, with the additional option of a ‘Not Applicable (N/A)’ response. The questionnaire has demonstrated the ability to highlight differences between normal-hearing individuals and CI users, as well as to correlate with QoL scores. The scores for each of the 18 frequency items and their corresponding importance items can be plotted on a matrix to help tailor individualized music training programs [10].

### 1.4. Need of a German Self-Report Measure for Music and Quality of Life

The MuRQoL questionnaire overcomes the limitations of previous music questionnaires designed for adult CI users and has the potential to be used as a screening tool to identify individual rehabilitation needs in clinical settings by measuring the impact of music on quality of life. Its clinical utility is supported by strong reliability, which exceeds the recommended criteria for individual-level measurements [10].

At present, the MuRQoL questionnaire, originally developed in English [24], has been translated and validated into Turkish [25], Italian [26], and, more recently, Spanish [27].

In order to promote a unified approach to research and clinical evaluation, working in a bilingual area of Italy, where Italian and German populations coexist, we decided to translate and validate the MurQol into German.

The present work was aimed to adapt and validate the Music hearing-related questionnaire (MuRQoL) into German to be used for comparisons across populations divided by language or culture.

## 2. Materials and Methods

### 2.1. Translation of the MuRQoL into German

The translation of the “Music related quality of life questionnaire” (MuRQoL) into the German version “Fragebogen zu Musik und Lebensqualität” (MuRQoL-De) was done, after receiving permission from its original designer [28], according to the recent international guidelines for translating and adapting hearing-related questionnaires for different languages and cultures and following the step-by-step guide [29]. The full German version of the questionnaire is provided in the Supplementary Materials (File S1).

After the preparation steps, the original questionnaire was translated from English into German by two native German bilingual translators. The two translators independently produced forward translations from English into German. A third team member compared the two versions, and discrepancies were discussed in consensus meetings until agreement was reached, resulting in a consolidated draft. This version was then back-translated into English by another bilingual translator unfamiliar with the original questionnaire. Any divergences between the back translation and the source version were carefully reviewed and resolved.

The process was supervised by a multidisciplinary team consisting of bilingual translators, clinicians with expertise in audiology and cochlear implantation, and a linguist. Special attention was given to ambiguous or potentially unclear items to ensure both semantic accuracy and cultural appropriateness. For example, the original English item “Can you hear differences in musical tone (i.e., how high or low music is)?” was adapted into German as “Können Sie in der Tonleiter die unterschiedlichen Töne erkennen? (z.B. hohe und tiefe)”. This wording was chosen to ensure clarity for German speakers by making the concept of pitch explicit through the reference to the musical scale.”

Finally, the pre-final version was piloted in a small group of CI users and NH participants (*n* = 10) to confirm comprehensibility and acceptability. Feedback indicated that the items were clear and culturally appropriate, and only minor wording adjustments were required.

### 2.2. Participants

There is no consensus in the literature regarding the ideal sample size for a focus group. More important than the sample size itself are the amount and richness of the data generated, as well as the appropriateness of the sample composition in relation to the research question [30,31].

For the present study, the minimum required sample size was estimated based on the maximum variance reported in the original version of the study scale [10], assuming a 95% confidence interval. Given an approximate variance of 16, a population size of 100, and a 50% response distribution, the estimated minimum sample size was 80 individuals.

The final sample of 75 participants was considered sufficient for an initial validation given the volume of data collected and the demographic diversity of both cochlear implant (CI) users and normal-hearing (NH) participants. While this sample size is adequate to support analyses of reliability and construct validity, larger cohorts will be needed in future studies to enable more detailed subgroup comparisons and to confirm the stability of the factor structure.

No specific selection criteria were applied to participant recruitment, except for proficiency in the German language and the absence of concurrent visual or cognitive impairments that could interfere with task performance. The NH participants were recruited from among relatives and friends of CI users and did not have a formal musical academic background; the unilaterally implanted subjects included were CI users only to avoid the potential confounding variable of bimodal stimulation (i.e., the use of a contralateral hearing aid).

Of the total sample, 13 CI users and 20 NH participants indicated that they have received previous musical education (for example: ability to play an instrument).

The study was conducted in accordance with the principles of the Helsinki Declaration [32] and approved by the Comitato Etico per la Sperimentazione Clinica della Provincia Autonoma di Bolzano (Parere nr. 1-2026). Data were examined in compliance with Italian privacy and sensitive data laws. All NH subjects and CI users were asked to fill in the final version of the questionnaire and an Information Form after obtaining their informed consent. Through the Information Form, demographic and clinical data (age, gender, etiology of deafness, CI model and years of CI use) were recorded. These data are summarized in Table 2.

### 2.3. Data Collection

The original design of the questionnaire, which allows participants to complete it independently either on paper or online, was retained in the German version. The final version of the MuRQoL-De was completed either in person during audiology consultations or in a self-administered format. The Information Form was administered at the same time and in the same manner.

For the MuRQoL-De, participants were excluded from the present analysis if they left any item blank or had more than three “not applicable” (N/A) responses across the entire questionnaire (*n* = 2).

NH participants were asked to provide a subjective audiometric self-assessment to determine their perceived hearing quality; ratings between 8 and 10 were considered indicative of “good hearing”.

Subjective hearing ability was collected only from NH participants as a brief self-assessment to exclude unrecognized hearing difficulties. We did not administer the same question to CI users because self-ratings in this group may be unreliable or systematically underestimated, particularly in individuals with profound or congenital deafness who lack prior experience of normal hearing. For CI participants, auditory ability was instead characterized by clinical audiometric data which provide more objective and reproducible measures.

All CI users, regardless of age of onset (pre- or postlingual deafness), demonstrated comparable auditory performance. Specifically, they achieved full open set spoken language understanding, defined as the ability to correctly recognize and repeat words or sentences without contextual or visual cues when presented in an unrestricted response format. In addition, audiometric test results obtained either at the time of questionnaire completion or during the most recent prior evaluation were considered. Only participants with a pure-tone average (PTA4: 0.5, 1, 2, and 4 kHz) of ≤35 dB HL were included in the study.

### 2.4. Data Analysis

Standard univariate analyses were conducted to identify potential differences in each recorded socio-demographic and clinical variable between NH individuals and CI users. Chi-squared tests were applied to categorical variables, while t-tests and Kruskal–Wallis tests were used for normally and non-normally distributed continuous variables, respectively. The distribution of each continuous variable was preliminarily assessed using the Shapiro–Wilk test. Item discrimination indices (corrected item–total correlations) were calculated, with values above 0.20 considered acceptable (Table 2).

The internal consistency of the MuRQoL questionnaire was evaluated by computing Cronbach’s alpha coefficients for the total Frequency and Importance scales, as well as for each subdomain (i.e., Music Perception and Music Engagement subscales); values above 0.70 were deemed acceptable (Table 3).

Construct validity was assessed by comparing the median values of the total scales and each subscale between NH individuals and CI users, using the Kruskal–Wallis test due to the non-parametric distribution of the data. A *p*-value < 0.05 was considered statistically significant [33,34] (Table 4).

Finally, crude regression coefficients were calculated to explore associations between each MuRQoL total scale and selected socio-demographic and clinical variables (Table 5).

All analyses were performed using Stata version 13.1 (Stata Corp, College Station, TX, USA, 2014) [35], with statistical significance set at a two-sided *p*-value < 0.05.

## 3. Results

We recruited two groups of participants for those mean age (M) and its standard deviation (SD) is reported: 32 (18 female and 14 male) CI users aged between 16 and 88 years (M = 41,0 years, SD = 23.7) and 43 (31 female and 12 male) NH subjects aged between 23 and 83 years (M = 48.9 years, SD = 16.1). The group of CI users consisted of both prelingually and postlingually deaf individuals. The majority of the participants (29 subjects) had been implanted with a MED-EL CI system (MED-EL GmbH, Innsbruck, Austria) and a small sample (3 subjects) with a COCHLEAR CI system (COCHLEAR Limited, Sydney, Australia), either unilaterally (50%) or bilaterally. Additionally, they had a minimum of 10 active electrodes in the case of a Medel CI system and of 22 in the case of a Cochlear CI system; they had at least 36 months of CI experience and were attending regular follow-up controls.

The internal consistency of the MuRQoL-De questionnaire was assessed using Cronbach’s alpha coefficients and its confidence interval (Ci), computed separately for the Frequency and Importance scales and their respective subscales (Table 3).

For the Frequency scale, the overall Cronbach’s alpha was 0.93 (95% Ci: 0.73–0.99), indicating excellent internal consistency. The Music Perception subscale showed an alpha of 0.94 (95% Ci: 0.59–0.99), while the Music Engagement subscale yielded a value of 0.81 (95% Ci: 0.42–0.99), both reflecting good to excellent consistency.

Similarly, the Importance scale demonstrated an overall Cronbach’s alpha of 0.94 (95% Ci: 0.73–0.99). The Music Perception subscale had the highest reliability (α = 0.95, 95% Ci: 0.59–0.99), and the Music Engagement subscale showed good reliability with an alpha of 0.84 (95% Ci: 0.42–0.99).

All coefficients exceeded the commonly accepted threshold of 0.70, supporting the internal consistency of the instrument and its subdomains.

Results from the MuRQoL questionnaire (Table 4) revealed statistically significant differences between NH subjects and CI users in the Frequency scale.

Specifically, NH participants reported significantly higher median scores than CI users for the overall Frequency scale (median = 4.0, IQR: 3.67–4.33 vs. 3.67, IQR: 3.06–3.89; *p* = 0.010) and the Music Perception subscale (median = 4.32, IQR: 3.68–4.59 vs. 3.73, IQR: 3.36–3.91; *p* = 0.007). No statistically significant difference was observed for the Music Engagement subscale (*p* = 0.09), although the median score was slightly higher in NH individuals.

In contrast, no significant differences between groups were found on the Importance scale, either overall (NH: 3.94, IQR: 3.17–3.95; Ci: 3.83, IQR: 3.44–4.28; *p* = 0.5) or within the Music Perception and Music Engagement subscales (*p* = 0.2 and *p* = 0.6, respectively).

These findings suggest that while the perceived importance of music-related experiences is similar between NH adults and CI users, the frequency of these experiences—especially in terms of music perception—is reduced among CI users.

Crude linear regression analyses (Table 4) were conducted to assess associations between the total Frequency and Importance scores of the MuRQoL questionnaire and selected socio-demographic and clinical variables.

None of the investigated variables showed statistically significant associations with either of the two total scales, as all 95% confidence intervals included zero. However, some trends were observed.

For the Frequency scale, a 10-year increase in age was associated with a non-significant decrease in score (β = −0.10; 95% Ci: −0.22 to 0.02), while higher educational level showed a positive, although not statistically significant, association (β = 0.34; 95% Ci: −0.15 to 0.83). Similarly, having a musical background or regular listening habits (β = 0.33; 95% Ci: −0.55 to 1.21), and playing a musical instrument (β = 0.30; 95% Ci: −0.28 to 0.88), were both associated with higher Frequency scores, though not significantly.

For the Importance scale, the effect of a 10-year increase in years of CI use approached statistical significance, showing a negative association (β = −0.62; 95% Ci: −1.24 to 0.01). Higher educational level also showed a positive trend (β = 0.45; 95% Ci: −0.02 to 0.92), though again not reaching statistical significance.

Gender, congenital hearing loss, and daily CI usage hours were not meaningfully associated with either MuRQoL scale.

These findings suggest potential, but not conclusive, associations between musical quality-of-life perception and factors such as age, education, and musical experience, warranting further investigation in larger samples.

**Figure 1:**
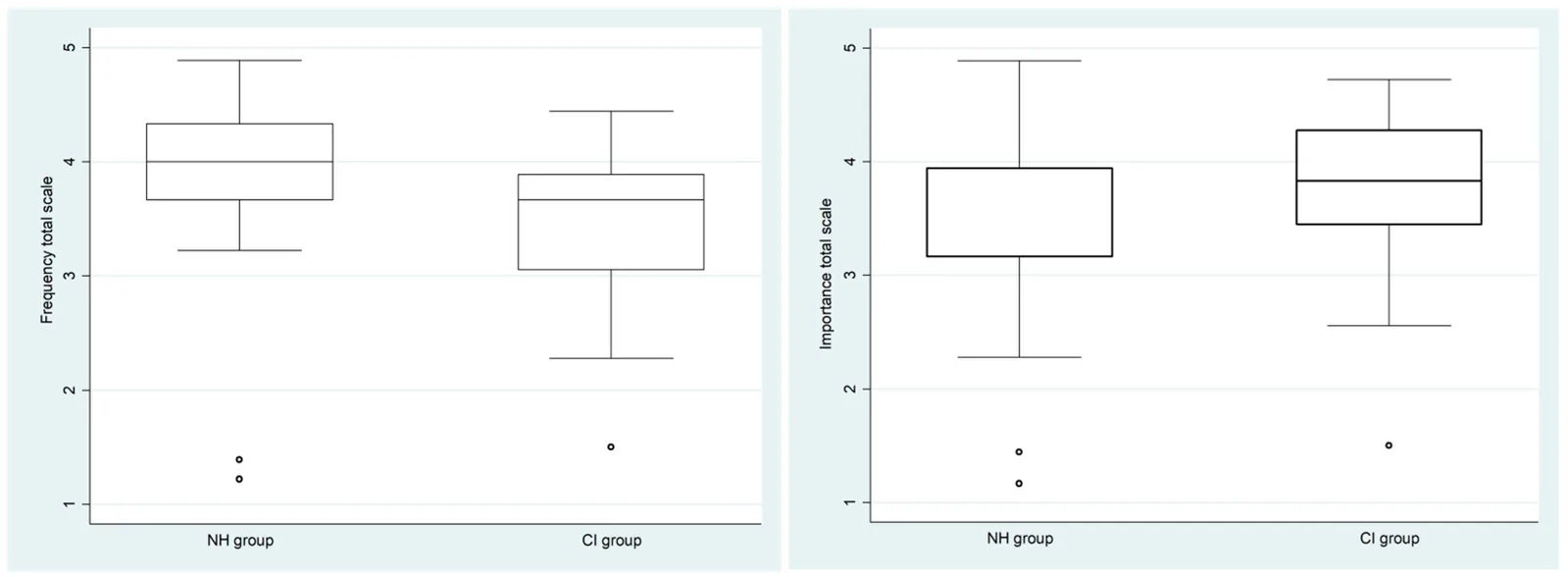
Boxplots of Frequency (top) and Importance (bottom) total scale for the normal hearing (NH) and the cochlear implant (CI) users’ group. The boxes represent the interquartile range (Q1 and Q3), with the median value reported as the inner, continuous line; the whiskers represent the top and the bottom 25% of the scores. Outlier values are shown as hollow circles.

Figure 1 presents boxplots illustrating the distribution of the total Frequency (top panel) and Importance (bottom panel) scores of the MuRQoL questionnaire, comparing individuals with normal hearing (NH) and cochlear implant (CI) users.

In each boxplot, the central box denotes the interquartile range (IQR), with the horizontal line inside the box indicating the median score. The whiskers extend to the minimum and maximum values within 1.5 times the IQR from the lower and upper quartiles, respectively. Outliers—defined as data points falling outside the whiskers—are displayed as hollow circles.

The plots show that the NH group tends to have higher median scores and less variability in both scales compared to the CI users’ group, particularly for the Frequency scale. These graphical trends support the statistical results reported in Table 4.

## 4. Discussion

The present study aimed to translate and validate the German version of the Music-Related Quality of Life Questionnaire (MuRQoL-De), maintaining the original structure and psychometric rigor. The findings confirm that the German version reliably captures the two core dimensions defined in the original instrument: Frequency, referring to the extent of music-related experiences, and Importance, reflecting the perceived value of music in daily life.

The internal consistency of the MuRQoL-De was high across both total scales and subscales, with Cronbach’s alpha values exceeding the recommended threshold of 0.70. These results are consistent with those reported in the original English version by Dritsakis et al., supporting the reliability of the MuRQoL-De in assessing music-related quality of life in German-speaking adults.

Discriminant validity was partially confirmed: NH participants reported significantly higher scores than CI users on the Frequency scale, particularly in the Music Perception subdomain. This aligns with prior studies indicating that CI users face limitations in music perception and enjoyment. For example, validations of the MuRQoL in Italian, Turkish, and Spanish populations similarly reported reduced frequency of music engagement among CI users, while NH participants consistently scored higher on this dimension. However, no significant differences emerged between groups on the Importance scale, suggesting that CI users place similar value on music as NH individuals despite reduced musical experiences. This cross-cultural consistency supports the notion that, although CI users may experience limitations in music perception, the subjective importance of music remains comparable to that of NH individuals across different populations.

Moreover, this dissociation between access to music and its perceived relevance points to a potential unmet need in auditory rehabilitation programs. Clinically, this highlights the potential benefit of rehabilitation strategies that integrate music listening or music therapy, which could help CI users translate their preserved motivation into greater real-world engagement. 

Crude regression analyses revealed no statistically significant predictors of either Frequency or Importance scores among the socio-demographic and clinical variables examined. Although the CI users sample included both pre- and postlingually deaf individuals, all participants demonstrated functional speech perception equivalence, as evidenced by full open set understanding and a free-field PTA of ≤35 dB HL. This uniformity in speech comprehension minimized the variability that might otherwise arise from differences in age of onset and supports the validity of analyzing the CI cohort as a single group in the present study. Nonetheless, some trends (such as a slight decline in Frequency with increasing age, and a possible negative association between years of CI use and the Importance scale) may warrant further exploration in future studies with larger samples.

These findings are in line with previous cross-cultural validations. The Italian [26], Turkish [25], and Spanish [27] versions all confirmed the MuRQoL’s applicability across different languages and cultural contexts. Our data further support this, affirming the MuRQoL’s cross-cultural robustness and suitability for German-speaking populations.

Although the sample size of this study was relatively limited (*n* = 75), it proved adequate for the purposes of validating the German version of the questionnaire, thereby supporting the robustness of the instrument across languages and cultural contexts. At the same time, the sample size does not allow for more detailed correlational analyses between variables or for comprehensive subgroup comparisons. Future studies with larger and multicenter cohorts will therefore be needed to explore associations with demographic and clinical variables and permit more powerful subgroup and multivariable analyses. 

A methodological consideration is that subjective hearing self-assessments were not obtained from CI users. Such measures may in fact be biased or underestimated in individuals with profound or congenital deafness, who have no baseline of normal hearing for comparison. For this reason, we relied on clinical audiological outcomes to describe CI users’ hearing ability. Nevertheless, future studies should include objective audiometry also for NH participants, to rule out undetected impairments that could influence music perception and to ensure a more complete characterization of hearing status across both groups. At the same time, collecting subjective self-assessments from CI users could provide additional insights, particularly when analyzed in correlation with mu-sic-related quality of life measures, and might enrich the interpretation of individual experiences with music.

Participants’ feedback indicated that the questionnaire was well accepted, with minimal missing responses, which supports its face validity and ease of administration. This suggests that the items are both culturally appropriate and relevant for German-speaking respondents.

In summary, the results confirm the construct validity and internal reliability of the MuRQoL-De and underline its relevance as a clinical and research tool for evaluating music-related quality of life in CI user populations.

A limitation of the present study is the absence of test–retest reliability data. For a clinical instrument, stability over time is essential; we therefore plan a subsequent study to administer the MuRQoL-De to a subsample of participants after an interval of 6 months and to compute intraclass correlation coefficients (ICC) and test–retest correlations. Establishing temporal stability will be a priority for future validation work.

Item-level psychometrics (e.g., item-total correlations and discrimination) were not fully explored here but would be interesting to include in follow-up studies with larger samples. Such analyses could identify the most informative items for clinical screening or short-form versions of the questionnaire.

## 5. Conclusions

The German version of the MuRQoL questionnaire (MuRQoL-De) demonstrates good psychometric properties, with a stable two-factor structure, high internal consistency, and acceptable construct validity. It effectively distinguishes between NH individuals and CI users in terms of music-related participation, while also highlighting the shared importance attributed to music across groups.

These findings support the use of the MuRQoL-De as a reliable and culturally appropriate instrument for assessing the impact of music on quality of life among German-speaking adults. Its application is particularly relevant in clinical and rehabilitative settings, where it can inform personalized interventions aimed at enhancing musical engagement and overall well-being, especially for individuals with hearing impairment.

As previously emphasized by Dritsakis et al., the MuRQoL fills a critical gap in audiological and quality-of-life research by focusing on music listening, an area often overlooked in conventional hearing-related QoL tools.

Future research with larger, more diverse samples should explore longitudinal changes in music-related quality of life, as well as the potential benefits of auditory training or music-based rehabilitation in CI users.

## Figures and Tables

**Table 1:** Objective music perception tests.

Clinical Appreciation of Music Perception (CAMP; University of Washington)
Appreciation of Music in Cochlear Implantees (AMICI; Columbia University)
Musical Sounds in Cochlear Implants perception test (MuSIC; Technical University of Munich)
Montreal Battery of Evaluation of Amusia (MBEA; University of Montreal)
Primary Measures of Music Audiation (PMMA, Temple University)
Multiple Stimulus with Hidden Reference and Anchor for Cochlear Implants (CI-MUSHRA; John Hopkins University)
Music EAR (University of Toronto)
Iowa Music Perception and Appraisal Battery—Children’s Version (IMPAB-C; University of Iowa)
Music in Children with Cochlear Implants (MCCI; Johns Hopkins University)
Subjective music assessment questionnaires:
Iowa Musical Background Questionnaire (IMBQ; University of Iowa). 10 × 10 ^19^
Music-Related Quality of Life Questionnaire (MuRQoL; University of Southampton). 20 × 10^26^
Munich Music Questionnaire (MuMu; Technical University of Munich)
University of Canterbury Music Listening Questionnaire (UCMLQ; University of Canterbury)

**Table 2:** Characteristics of the sample, overall and by hearing status (CI—cochlear implant—users versus subjects with NH—normal hearing).

Variables	Total Sample (N = 75)	CI Users (N = 32)	NH Subjects (N = 43)	*p* *
Gender (%): male, female, nonbinary	34.7; 65.3; 0	27.9; 72.1; 0	43.8; 56.2; 0	0.2
Age:				
– mean age in years (SD)	45.5 (2.3)	41.0 (23.7)	48.9 (16.1)	0.09
– ≥65 years, %	17.3	21.9	14.0	0.4
Educational level, %				<0.001
– Primary/lower secondary school	25.3	53.1	4.7	
– Diploma	60.0	40.6	74.4	
– University degree or higher	14.7	6.3	20.9	
Hearing capacity **				
– mean value (SD)	--	--	9.0 (1.3)	--
– score < 8, %	--	--	11.6	--
Congenital hypoacusia, %	--	50.0	--	--
Mean age of hypoacusia onset (SD)	--	10.2 (5.6)	--	--
Median age of CI implantation—right (IQR)	--	4.5 (1.0–27.0)	--	--
Median age of CI implantation—left (IQR)	--	4.0 (1.0–20.0)	--	--
Median duration of CI use in years (IQR)	--	10.0 (3.0–17.0)	--	--
Mean use of CI in hours (SD)	--	14.8 (2.3)	--	--
Use of LIS, %	--	6.3	--	--
Speechtherapy/hearing training, %	--	93.8	--	--
Musical knowledge, % ***	44.0	40.6	46.5	0.6
Musical background or habitual listening habits, %	88.0	72.7	100	0.003

* Chi-squared test for categorical variables; *t*-test and Kruskal–Wallis test for parametric and non-parametric continuous variables, respectively. ** Assessed through self-evaluation on a 10-point scale, from 1 (lowest capacity) to 10 (highest capacity). A value < 7 is suggestive of impaired hearing capacity. *** Including self-reported musical knowledge or ability to play an instrument. SD: standard deviation. IQR: interquartile range.

**Table 3:** MuRQoL questionnaire internal consistency (Cronbach’s alpha).

Items	Cronbach’s Alpha (95% Ci) (N = 75)
1. Frequency scale	
Overall	0.93 (0.73–0.99)
Music perception subscale	0.94 (0.59–0.99)
Music engagement subscale	0.81 (0.42–0.99)
2. Importance scale	
Overall	0.94 (0.73–0.99)
Music perception subscale	0.95 (0.59–0.99)
Music engagement subscale	0.84 (0.42–0.99)

Ci: confidence interval.

**Table 4:** Results of the MuRQoL Questionnaire in the selected sample, by hearing status.

Items	NH Subjects (N = 43)	CI Users (N = 32)	*p* *
1. Frequency scale, median value (IQR)			
Overall	4.0 (3.67–4.33)	3.67 (3.06–3.89)	**0.010**
Music perception subscale	4.32 (3.68–4.59)	3.73 (3.36–3.91)	**0.007**
Music engagement subscale	3.57 (3.14–3.86)	3.29 (2.43–3.71)	0.09
2. Importance scale, median value (IQR)			
Overall	3.94 (3.17–3.95)	3.83 (3.44–4.28)	0.5
Music perception subscale	4.18 (3.27–4.19)	4.09 (3.73–4.55)	0.2
Music engagement subscale	3.57 (2.71–3.86)	3.57 (2.86–4.14)	0.6

* Kruskal–Wallis test. Statistically significant differences between groups (*p* < 0.05) are highlighted in bold. IQR: interquartile range. NH: normal hearing. CI: cochlear implant.

**Table 5:** Crude regression coefficients between each MuRQoL questionnaire total scale and selected socio-demographic and clinical variables.

Variables	Frequency Scale Crude Coeff. (95% Ci)	Importance Scale Crude Coeff. (95% Ci)
Gender (male)	−0.10 (−0.69; 0.48)	−0.38 (−0.94; 0.18)
Age, 10-year increase	−0.10 (−0.22; 0.02)	−0.02 (−0.14; 0.11)
Educational level, 1-category increase *	0.34 (−0.15; 0.83)	0.45 (−0.02; 0.92)
Musical background or habitual listening habits, yes vs. no	0.33 (−0.55; 1.21)	−0.06 (−0.71; 0.58)
Musical knowledge (playing instruments), yes vs. no	0.30 (−0.28; 0.88)	−0.02 (−0.60; 0.57)
Congenital hypoacusia, yes vs. no	0.27 (−0.30; 0.85)	0.02 (−0.56; 0.60)
Years of CI use, 10-year increase	−0.12 (−0.72; 0.48)	−0.62 (−1.24; 0.01)
Hours of CI use, 1 h increase	0.06 (−0.13; 0.14)	0.04 (−0.09; 0.16)

Coeff.: coefficient; Ci: confidence interval; CI: cochlear implant * Educational level was categorized into: 1. primary or lower secondary school; 2. high school diploma; 3. university degree or higher.

## Data Availability

The data that support the findings of this study are available from the corresponding author (N.G.), upon reasonable request.

## Supplementary Materials

The following supporting information can be downloaded at: https://www.mdpi.com/article/10.3390/audiolres16040120/s1, File S1: Questionnaire on “Music and Quality of Life” Part I and Part II.
