# Supplementary material for: The Music-Related Quality of Life (MuRQoL) Questionnaire: Adaptation and Validation into the German Fragebogen zu Musik und Lebensqualität
Source: Audiol Res. 2026 Aug 17;16(4):120. doi: 10.3390/audiolres16040120 (PMC13509632; doi:10.3390/audiolres16040120)
Supplement: Supplementary file 1 [file audiolres-16-00120-s001.zip › File S1. The_MuRQoL_questionnaire_v2 Tedesco DEF.pdf]

Fragebogen zu “Musik und Lebensqualität“

Part I

Der erste Teil des Fragebogens beschäftigt sich mit der Einstellung zu Musik und die Fähigkeit diese wahrzunehmen, dem Hören von Musik in verschiedenen Lebensbereichen und wie oft man sich mit Musik beschäftigt.
Bitte beantworten Sie die Fragen mit den 6 Ihnen zur Verfügung stehenden Antwortmöglichkeiten:
1: nie, 2: selten, 3: manchmal, 4: oft, 5: immer, KA: keine ahnung.

	1	2	3	4	5	KA
**WAHRNEHMUNG VON MUSIK**						
1. Können Sie beim Hören von Musik unterschiedliche Rhythmen erkennen?	☐	☐	☐	☐	☐	☐
2. Können Sie einer Melodie folgen? (z.b. der Melodie eines Liedes oder eines bekannten Stückes)	☐	☐	☐	☐	☐	☐
3. Können Sie in der Tonleiter die unterschiedlichen Töne erkennen? (z.B. hohe und tiefe)	☐	☐	☐	☐	☐	☐
4. Können Sie den Text in Liedern verstehen?	☐	☐	☐	☐	☐	☐
5. Können Sie Musikinstrumente an ihrem Klang unterscheiden? (Violine, Klavier, Saxophon, Gitarre…)	☐	☐	☐	☐	☐	☐
6. Können Sie die das Emotionale einer Musik erkennen? (z.B. welches Gefühl sie vermitteln will?)	☐	☐	☐	☐	☐	☐
7. Können Sie die Musik mühelos hören, ohne sich konzentrieren zu müssen?	☐	☐	☐	☐	☐	☐
8 Können Sie bekannte Musik erkennen? (z.B. ein Lied, einen Sänger oder eine Melodie)	☐	☐	☐	☐	☐	☐
9. Können Sie die Qualität der erbrachten Leistung beurteilen? (z.B. die des Gesungenen oder die des instrumentalen Anteils)	☐	☐	☐	☐	☐	☐
10. Sind Sie der Meinung, Musik so wie alle anderen zu hören?	☐	☐	☐	☐	☐	☐
11. Klingt die Musik für Sie abgestimmt (wohlklingend, melodisch)?	☐	☐	☐	☐	☐	☐
**BESCHÄFTIGUNG MIT MUSIK**						
12. Gefällt es Ihnen Musik in lauter Umgebung zu hören (z.B. Partys, Restaurants, im Auto) ohne dabei auch visuell stimuliert zu werden?	☐	☐	☐	☐	☐	☐
13. Gefällt es Ihnen Musik im Fernseher, über DVD, Handy oder Computer zu hören und gleichzeitig visuell stimuliert zu sein?	☐	☐	☐	☐	☐	☐
14. Genießen Sie musikalischen Hintergrund, während Sie anderen Beschäftigungen nachgehen? (z.B. beim Lesen, Malen, Gärtnern, Putzen, Trainieren oder sich Entspannen)	☐	☐	☐	☐	☐	☐
15. Hören Sie Musik auch auf Reisen? (z.B. im Auto)	☐	☐	☐	☐	☐	☐
16. Hören Sie auch Musik, die Sie noch nicht kennen?	☐	☐	☐	☐	☐	☐
17. Besuchen Sie Musikveranstaltungen? (z.B. Musicals, Konzerte, Opern, Festivals)	☐	☐	☐	☐	☐	☐
18. Singen Sie oder spielen Sie ein Musikinstrument? Pfeifen Sie, wenn Sie alleine sind?	☐	☐	☐	☐	☐	☐

Part II

Im zweiten Teil des Fragebogens, werden dieselben Fragen gestellt. In diesem Fall sollen Ihre Antworten Einblick geben, wie wichtig für Sie die Gabe Musik wahrzunehmen ist, sie anzuhören und die Beschäftigung mit Musik ist.
Bitte beantworten Sie die Fragen mit den 6 Ihnen zur Verfügung stehenden Antwortmöglichkeiten:
1: unwichtig, 2: nicht sehr wichtig, 3: wichtig, 4: sehr wichtig, 5: ganz besonders wichtig, KA: keine ahnung.

	1	2	3	4	5	KA
**WAHRNEHMUNG VON MUSIK**						
1. Wie wichtig ist Ihnen beim Hören von Musik unterschiedliche Rhythmen zu erkennen?	☐	☐	☐	☐	☐	☐
2. Wie wichtig ist Ihnen, einer Melodie folgen zu können (z.B. die Melodie eines Liedes oder der eines bekannten instrumentalen Stückes)?	☐	☐	☐	☐	☐	☐
3. Wie wichtig ist Ihnen unterschiedliche Töne in der Tonleiter zu erkennen (z.B. hohe und tiefe)?	☐	☐	☐	☐	☐	☐
4. Wie wichtig ist Ihnen den Text in Liedern zu verstehen?	☐	☐	☐	☐	☐	☐
5. Wie wichtig ist Ihnen, den Klang unterschiedlicher Instrumente zu erkennen (z.B. Violine, Klavier, Saxofon, Gitarre…)?	☐	☐	☐	☐	☐	☐
6. Wie wichtig ist Ihnen, das Emotionale einer Musik zu erkennen (z.B. welches Gefühl sie vermitteln will)?	☐	☐	☐	☐	☐	☐
7. Wie wichtig ist Ihnen, die Musik mühelos hören zu können, ohne sich konzentrieren zu müssen?	☐	☐	☐	☐	☐	☐
8. Wie wichtig ist Ihnen, vertraute Musik zu erkennen (z. B. ein Lied, einen Sänger oder eine Melodie)?	☐	☐	☐	☐	☐	☐
9. Wie wichtig ist Ihnen, die Qualität einer musikalischen Leistung zu beurteilen (z.B. die einer Singstimme oder eines instrumentalen Stückes)?	☐	☐	☐	☐	☐	☐
10. Wie wichtig ist Ihnen, Musik so wie alle anderen hören zu können?	☐	☐	☐	☐	☐	☐
11. Wie wichtig ist Ihnen, Musik klangvoll zu hören (wohlklingend, melodisch)?	☐	☐	☐	☐	☐	☐
**BESCHÄFTIGUNG MIT MUSIK**						
12. Wie wichtig ist Ihnen, Musik in lauter Umgebung zu hören (z.B. Partys, Restaurants, im Auto) ohne dabei auch visuell stimuliert zu werden?	☐	☐	☐	☐	☐	☐
13. Wie wichtig ist Ihnen, Musik im Fernseher, über DVD, Handy oder Computer zu hören und gleichzeitig auch visuell stimuliert zu sein?	☐	☐	☐	☐	☐	☐
14. Wie wichtig ist Ihnen, mit musikalischem Hintergrund anderen Tätigkeiten nachzugehen (z.B. beim Lesen, Malen, Gärtnern, Säubern, Trainieren oder sich Entspannen)?	☐	☐	☐	☐	☐	☐
15. Wie wichtig ist Ihnen, Musik auf Reisen zu hören (z.B. im Auto)?	☐	☐	☐	☐	☐	☐
16. Wie wichtig ist Ihnen, neue, noch unbekannte Musik zu hören?	☐	☐	☐	☐	☐	☐
17. Wie wichtig ist Ihnen, Musikveranstaltungen zu besuchen? (z.B. Musicals, Konzerte, Opern, Festivals)	☐	☐	☐	☐	☐	☐
18. Wie wichtig ist Ihnen, zu singen oder ein Musikinstrument zu spielen? Oder zu pfeifen, wenn Sie alleine sind?	☐	☐	☐	☐	☐	☐
